# Identification of serum exosomal lncRNA MIAT as a novel diagnostic and prognostic biomarker for gastric cancer

**DOI:** 10.1002/jcla.23323

**Published:** 2020-04-10

**Authors:** Hao Xu, Jie Zhou, Jin Tang, Xuli Min, Tingting Yi, Jing Zhao, Yongjun Ren

**Affiliations:** ^1^ Department of Interventional Radiology Affiliated Hospital of North Sichuan Medical College Nanchong China; ^2^ Department of Hepatobiliary Surgery Affiliated Hospital of North Sichuan Medical College Nanchong China; ^3^ Department of Gastrointestinal Surgery Affiliated Hospital of North Sichuan Medical College Nanchong China; ^4^ Department of Laboratory Affiliated Hospital of North Sichuan Medical College Nanchong China

**Keywords:** diagnosis, gastric cancer, LncRNA MIAT, prognosis

## Abstract

**Background:**

Accumulating evidence has demonstrated that long non‐coding RNAs (lncRNAs) MIAT is significantly upregulated in many cancer types including gastric cancer (GC). However, the potential clinical significance of serum exosomal MIAT in GC is unknown.

**Methods:**

In this study, a total of 109 GC patients, 48 gastric adenoma patients, and 50 healthy individuals were recruited. Serum exosomal MIAT levels were detected in all participants using quantitative real‐time reverse transcription‐polymerase chain reaction (qRT‐PCR).

**Results:**

The exosomes we extracted from the serum samples were positive for TSG101, CD63, and Flotillin‐1, which were known exosome markers. Serum exosomal MIAT levels were significantly higher in GC patients than in gastric adenoma patients and healthy controls. Interestingly, gastric adenoma patients with higher serum exosomal MIAT expression were more prone to develop GC. In addition, serum exosomal MIAT levels were significantly decreased in post‐treatment blood samples compared to pre‐treatment samples, while markedly increased in the cases suffering recurrence. Moreover, serum exosomal MIAT upregulation was significantly associated with worse clinical variables and shorter survival. Furthermore, serum exosomal MIAT was identified as an independent prognostic factor for GC.

**Conclusions:**

Collectively, serum exosomal lncRNA MIAT might serve as a promising novel biomarker for monitoring the progression of GC.

## 1 INTRODUCTION

Gastric cancer (GC) is the fourth most frequent cancer and one of the most leading causes of cancer‐related mortality around the world.^1^ In 2018, approximately 1 000 000 new cases of GC and 783 000 deaths were reported worldwide.^2^ Unfortunately, most of GC patients are diagnosed at the late stages, frequently with invasion or metastasis. Despite considerable progress has been achieved in the treatment of GC, survival for patients with unresectable advanced or recurrent GC is dismal.^3^ Therefore, identification of novel biomarkers for early diagnosis and prognosis prediction of GC is urgently required.

Long non‐coding RNAs (lncRNAs) are a class of non‐coding RNAs longer than 200 nucleotides and lack protein‐coding capacity.^4^ LncRNAs have been found to play important roles in a variety of biological processes such as chromatin remodeling, cell differentiation, cell growth, and proliferation.^5^, ^6^ Emerging evidence has demonstrated that lncRNAs are closely involved in GC tumorigenesis and progression. For instance, Yan et al revealed that lncRNA SNHG6 was upregulated both in GC tissues and cell lines. Downregulation of SNHG6 significantly suppressed the malignant behaviors of GC cells, indicating SNHG6 played an oncogenic role in GC.^7^ The expression level of lncRNA OLC8 was markedly upregulated in GC specimens and cell lines. In addition, suppression of lncRNA OLC8 inhibited the oncogenic activities of GC cells both in vitro and in vivo.^8^ The expression level of RP11‐555H23.1 was significantly decreased in GC tissues, and its reduction was closely correlated with tumor‐node‐metastasis (TNM) stage.^9^ Gao et al reported that lncRNA NBAT‐1 was significantly downregulated in GC tissues, and NBAT‐1 overexpression markedly decreased the proliferative capacity of cancer cells, suggesting that NBAT‐1 might act as a tumor suppressor in GC.^10^

Exosomes are membrane vesicles and can be found in serum, plasma, urine, and other body fluids.^11^ LncRNAs are found in circulating exosomes and might be used as candidate biomarkers for the detection and prognosis prediction of cancers.^12^ For instance, the expression of serum exosomal lncRNA PCSK2‐2:1 was significantly reduced in patients with GC, and downregulation of serum exosomal lncRNA PCSK2‐2:1 was associated with adverse clinical parameters of GC.^13^ Similarly, serum exosomal lnc‐GNAQ‐6:1 level was lower in GC patients and exhibited good performance for discriminating GC patients from healthy volunteers.^14^

Myocardial infarction associated transcript (MIAT) was first identified as a lncRNA in 2006 and mapped to human chromosome 12q12.1.^15^ MIAT was upregulated both in GC tissues and cell lines. In vitro and in vivo analyses revealed that MIAT downregulation significantly suppressed the oncogenic activities of GC cells.^16^, ^17^ However, the potential clinical value of circulating exosomal MIAT in GC remained unclear. In this study, we first detected serum exosomal MIAT levels in GC patients, and then, the correlations between serum exosomal MIAT expression and the prognosis of GC were further evaluated.

## 2 MATERIALS AND METHODS

### 2.1 Patients and serum collection

A total of 109 patients with GC, 48 patients with gastric adenoma, and 50 healthy volunteers were enrolled in this study. Of all 109 GC cases, 81 were males and 28 were females. None of these cases have received prior treatment before first‐time blood sampling. The pathological staging of GC was assessed according to the seventh edition of the Union for International Cancer Control (UICC) tumor‐node‐metastasis (TNM) staging system. The exclusion criteria for GC were as follows: (a) with a secondary malignancy, (b) presence of severe heart disease such as acute myocardial infarction, arrhythmia, and heart failure, and (c) presence of systemic diseases such as liver failure, multiple organ dysfunction syndrome, and chronic kidney disease. The clinical characteristics of GC patients were summarized in Table 1. No patient was lost to follow up. The follow‐up time of GC patients ranged from 2.3 to 60.0 months with average of 44.1 ± 20.3 months. A total of 5 mL venous blood was drawn from each participant. The blood samples were then centrifuged at 3000 g for 10 minutes at room temperature, and the supernatant was collected and stored at −80°C. This study was approved by the Ethics Committees of Affiliated Hospital of North Sichuan Medical College. Written informed consents were collected from all participants or their relatives.

**Table 1:** Relationship of serum exosomal lncRNA MIAT expression and clinical variables of GC

Variables	Total	Low lncRNA MIAT	High lncRNA MIAT	*P*‐value
Gender				
Male	81	43	38	.3507
Female	28	12	16	
Age (y)				
<60	56	30	26	.5041
≥60	53	25	28	
Tumor size (cm)				
<5	50	29	21	.1472
≥5	59	26	33	
Invasive depth				
T1/T2	65	36	29	.2112
T3/T4	44	19	25	
Distant metastasis				
Negative	93	50	43	.0962
Positive	16	5	11	
Differentiation				
Well/moderate	64	38	26	.0264
Poor	45	17	28	
Lymphatic metastasis				
Negative	35	26	9	.0006
Positive	74	29	45	
TNM stage				
I/II	44	34	10	<.0001
III/IV	65	21	44	

### 2.2 Isolation of exosomes

Exosomes were isolated from the serum samples using the ExoQuick Exosome Precipitation Solution (System Biosciences) according to the manufacturer's instructions. Briefly, followed by thawing on ice, the serum was centrifuged at 3000 g for 10 minutes and filtrated with a 0.22 μm syringe filter (Millipore) to eliminate possible cell debris. Then, the supernatant was mixed with 125 μL ExoQuick reagent, and the mixture was incubated at 4°C for 30 minutes. The exosomes were pelleted by centrifuging the mixture at 1500 g for 30 minutes. The pellets containing exosomes were resuspended in phosphate‐buffered saline (PBS) and stored at −80°C for further experiments.

### 2.3 Western blot

Protein samples (20 µg) were separated on 8%‐12% sodium dodecyl sulfate polyacrylamide gel and transferred onto polyvinylidene difluoride (PVDF) membranes. Followed by blocking with 5% non‐fat milk in TBST for 1 hour at room temperature, the membranes were probed with primary antibodies of CD63 (Abcam), TSG101 (Abcam), and Flotillin‐1(Abcam) in the cold room overnight. Followed by washing three times in TBST, the membranes were incubated with appropriated secondary antibodies for 1h at room temperature. The blots were visualized by SuperSignal™ West Pico PLUS Chemiluminescent Substrate (Thermo Fisher Scientific, Inc).

### 2.4 Total RNA extraction and quantitative real‐time reverse transcription‐polymerase chain reaction (qRT‐PCR)

Total RNA was isolated from serum samples using mirVana™ miRNA Isolation Kit (Ambion). Reverse transcription was performed with TaqMan MicroRNA Reverse Transcription Kit (Applied Biosystems). Amplification and detection of serum exosomal MIAT were performed using SYBR^®^ Premix Ex Taq™ II (Takara) with the 7500 real‐time RT‐PCR system (Applied Biosystems). The qRT‐PCR was performed under the following reaction condition: 95°C for 30 seconds, followed by 40 cycles of 95°C for 5 seconds and 60°C for 34 seconds. The relative serum exosomal MIAT expression was subsequently calculated using the 2^–ΔΔ^ *^C^* ^t^ method, and cel‐miR‐39 was used as an external control. Primers were listed as follows: MIAT forward: 5′‐GGACGTTCACAACCACACTG‐3′, MIAT reverse: 5′‐TCCCACTTTGGCATTCTAGG‐3′; cel‐miR‐39 forward: 5′‐CAGAGTC‐ACCGGGTGTAAAT‐3′, cel‐miR‐39 reverse:5′‐CCAGTGCGTGTCGTGGAGTC‐3′.

### 2.5 Statistical analysis

Statistical analyses were performed with GraphPad Prism 5.0 (GraphPad Software, La Jolla, CA, USA) and MedCalc 9.0 (MedCalc Software Inc). The difference of serum exosomal MIAT expression levels in different groups was analyzed by Kruskal‐Wallis test. The chi‐square test was performed to determine the associations between serum exosomal MIAT and the clinicopathological variables. Receiver operating characteristic (ROC) curve and the area under the curve (AUC) were used to assess the diagnostic accuracy of serum exosomal MIAT. Overall survival (OS) was defined as the time from diagnosis until the date of death or last follow‐up. Relapse‐free survival (RFS) was defined as the time from diagnosis until the date of relapse or death or last follow‐up. OS and RFS rates were calculated by the Kaplan‐Meier method with the log‐rank test. Multivariate Cox regression analysis was used to assess the independent prognostic factors for OS *P* value <.05 was considered statistically significant.

## 3 RESULTS

### 3.1 Serum exosomal MIAT was upregulated in GC

**Figure 1:**
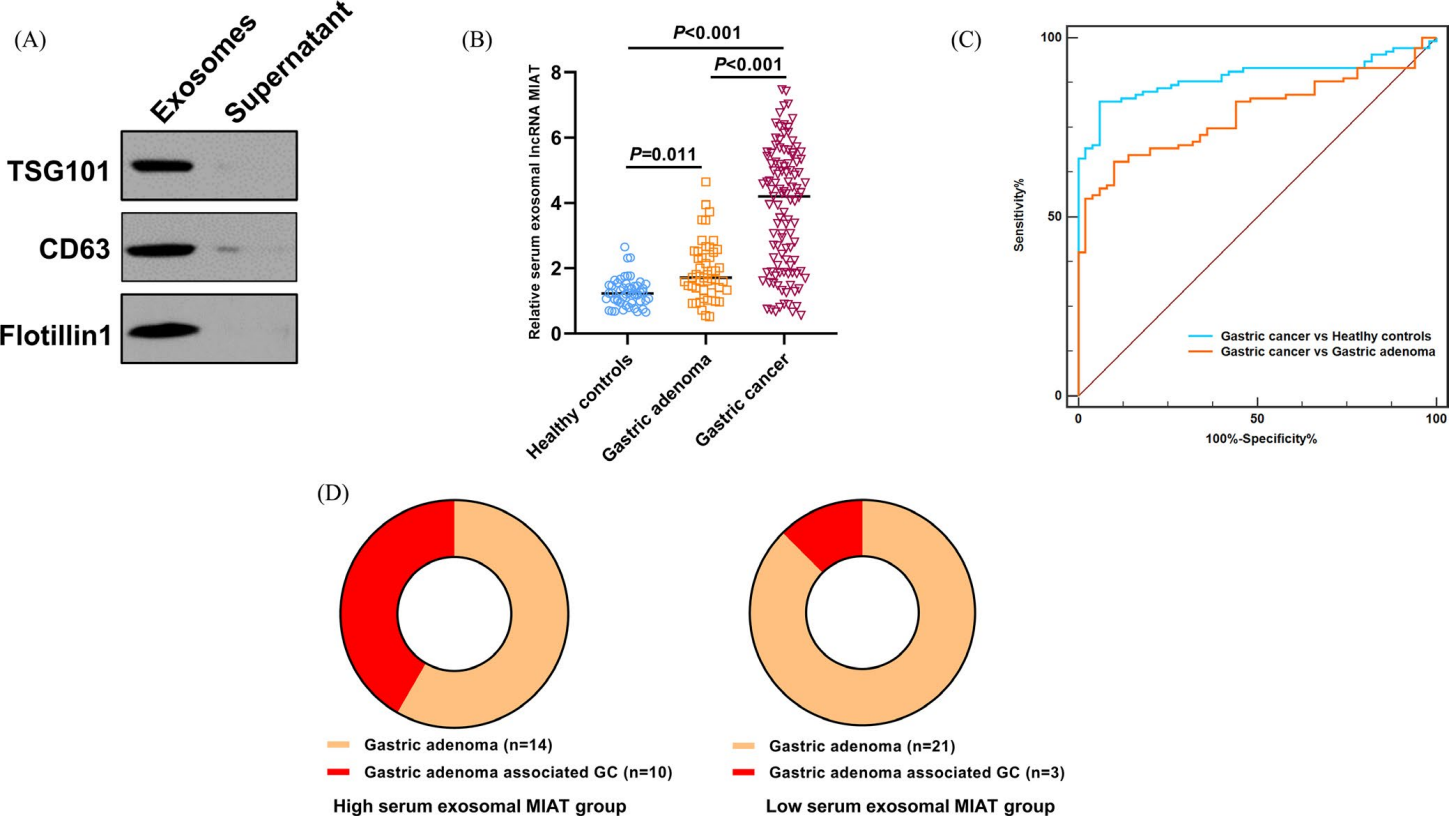
Serum exosomal MIAT levels were upregulated in GC. A, Our Western blot results demonstrated that the exosomes we extracted from the serum samples were positive for exosome markers including TSG101, CD63, and Flotillin‐1. B, Serum exosomal MIAT levels were significantly higher in GC patients than in gastric adenoma patients and healthy controls. C, ROC analysis showed that serum exosomal MIAT well‐identified GC patients from healthy controls and gastric adenoma patients. D, Gastric adenoma patients with higher serum exosomal MIAT expression were more prone to progress into GC

Our Western blot results demonstrated that the exosomes we extracted from the serum samples were positive for exosome markers including TSG101, CD63, and Flotillin‐1. However, no or weak signal was detected in the supernatant samples (Figure 1A). As shown in Figure 1B, the expression level of serum exosomal MIAT was markedly higher in GC patients than in gastric adenoma patients (*P* < .001) and healthy controls (*P* < .001). In addition, serum exosomal MIAT level was higher in patients with gastric adenoma than in healthy volunteers (*P* = .011). Moreover, ROC curve analysis revealed that serum exosomal MIAT well‐discriminated GC patients from healthy controls (AUC = 0.892) and GC patients from gastric adenoma patients (AUC = 0.787, Figure 1C).

All gastric adenoma patients were divided into two subgroups using the median value of serum exosomal MIAT levels. As presented in Figure 1D, gastric adenoma patients in the high serum exosomal MIAT subgroup suffered a significantly higher risk to progress into GC (10/24) than those in the low serum exosomal MIAT subgroup (3/24)**.**

### 3.2 High serum exosomal MIAT was correlated with aggressive clinical variables

**Figure 2:**
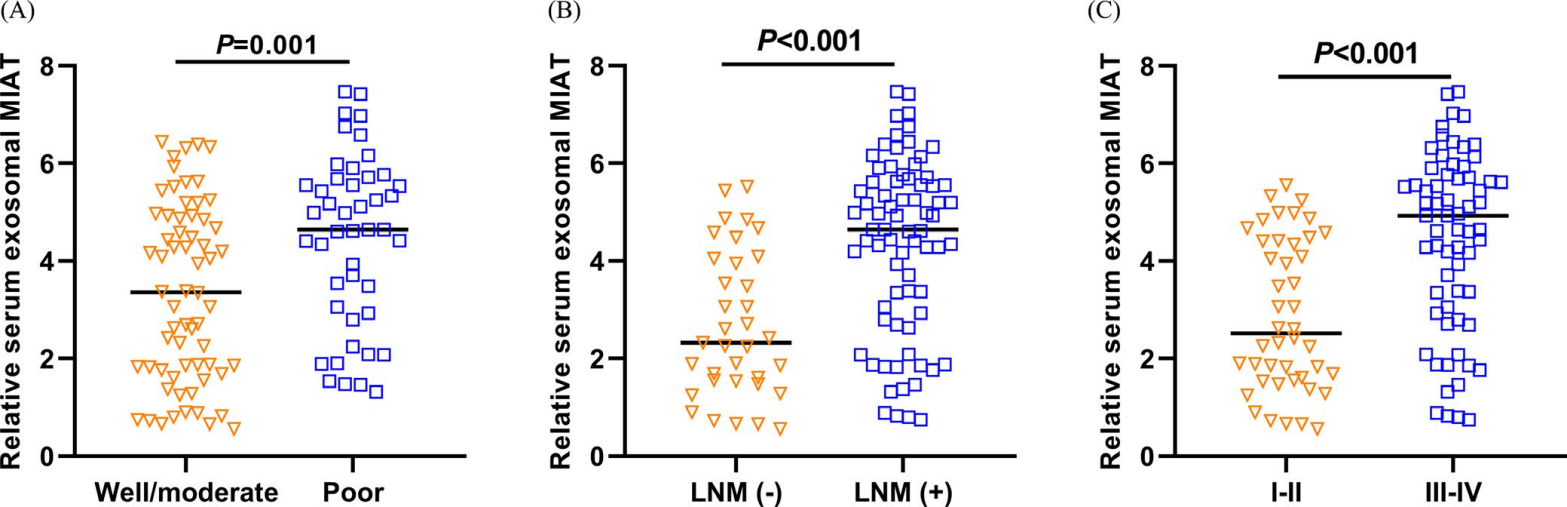
High serum exosomal MIAT was correlated with aggressive clinical variables. A, Increased serum exosomal MIAT levels were found in GC patients with poorly differentiated tumor grade. B, The GC patients with positive lymph node metastasis had higher serum exosomal MIAT levels. C, The GC patients at the advanced stage had higher serum exosomal MIAT levels

Then, the associations between serum exosomal MIAT levels and clinical variables were further investigated, and the median value of serum exosomal MIAT levels was used to classify all GC patients into high serum exosomal MIAT expression group (n = 54) and low serum exosomal MIAT expression group (n = 55). The results demonstrated that high serum exosomal MIAT was strongly associated with differentiation (*P* = .0264), lymphatic metastasis (*P* = .0006), and TNM stage (*P* < .0001). However, there were no significant associations of serum exosomal MIAT expression with gender (*P* = .3507), age (*P* = .5041), tumor size (*P* = .1472), invasive depth (*P* = .2112), and distant metastasis (*P* = .0962) (Table 1). In addition, GC patients with poorly differentiated tumor grade (*P* = .001), or with lymph node metastasis (*P* < .001), or at the advanced stages (*P* < .001) had significantly higher serum exosomal MIAT levels than those with well/moderate differentiated tumor grade, or without lymph node metastasis, or at the early stages (Figure 2A‐2C).

### 3.3 Changes in serum exosomal MIAT level before and after treatment

**Figure 3:**
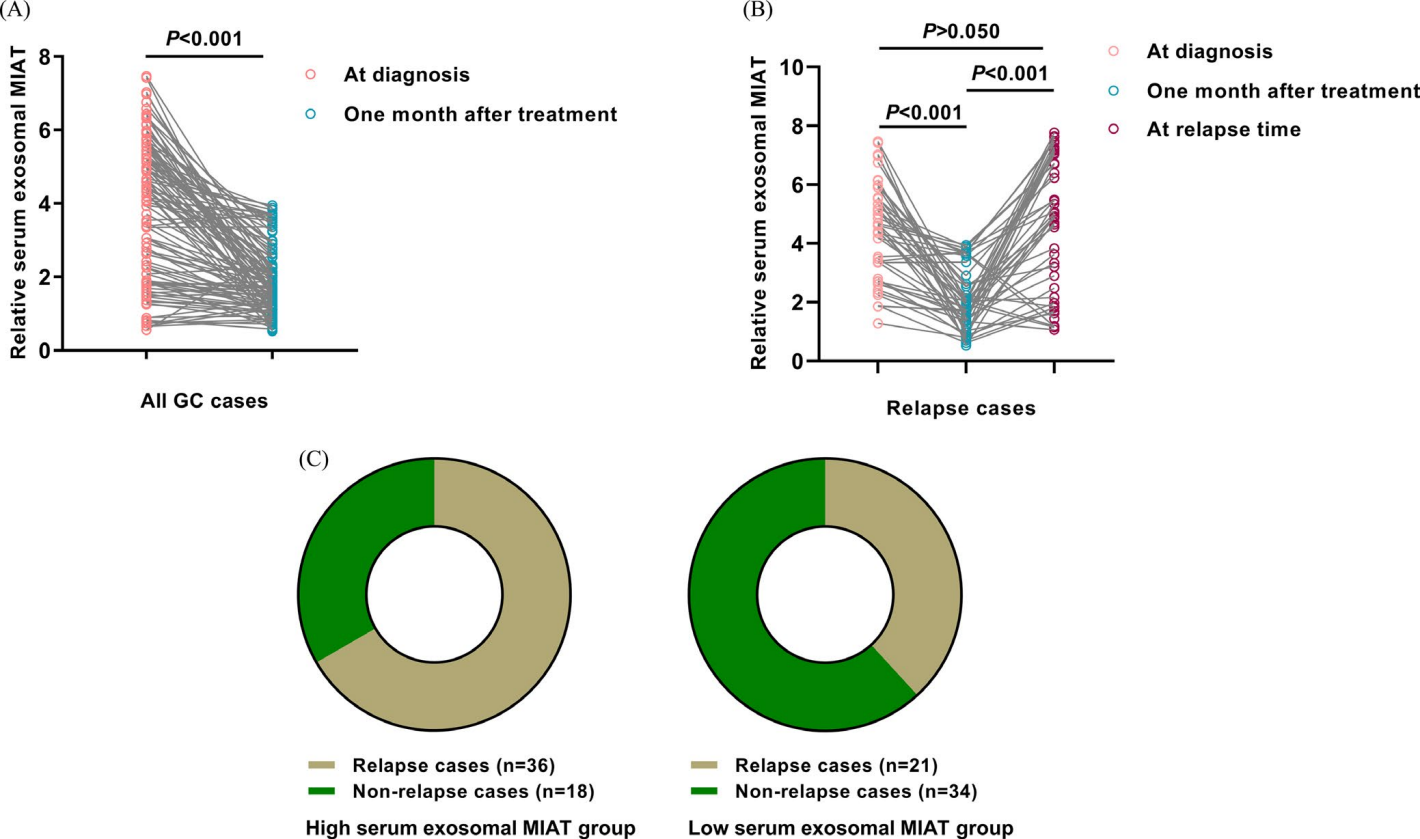
Changes in serum exosomal MIAT level before and after treatment. A, Serum exosomal lncRNA MIAT levels were significantly decreased one month after treatment. B, For patients with recurrence, serum exosomal MIAT levels were significantly decreased one month after treatment, while markedly increased when experiencing relapse. C, GC patients with higher serum exosomal lncRNA MIAT expression had higher risk of recurrence

The serum exosomal MIAT levels in paired blood samples from all 109 GC patients were compared before and 1 month after treatment. As showed in Figure 3A, serum exosomal MIAT levels were significantly decreased in the post‐treatment samples (*P* < .001). A total of 57 cases relapsed during the follow‐up. Interestingly, serum exosomal MIAT levels were also markedly downregulated in these relapsed cases one month after treatment. However, the levels of serum exosomal MIAT were significantly increased at the timepoint of relapse. (*P* < .001, Figure 3B). As presented in Figure 3C, GC patients in the high serum exosomal MIAT subgroup had a higher recurrence rate (36/54) than those in the low serum exosomal MIAT subgroup (21/55).

### 3.4 GC patients with higher serum exosomal MIAT suffered shorter OS and RFS

**Figure 4:**
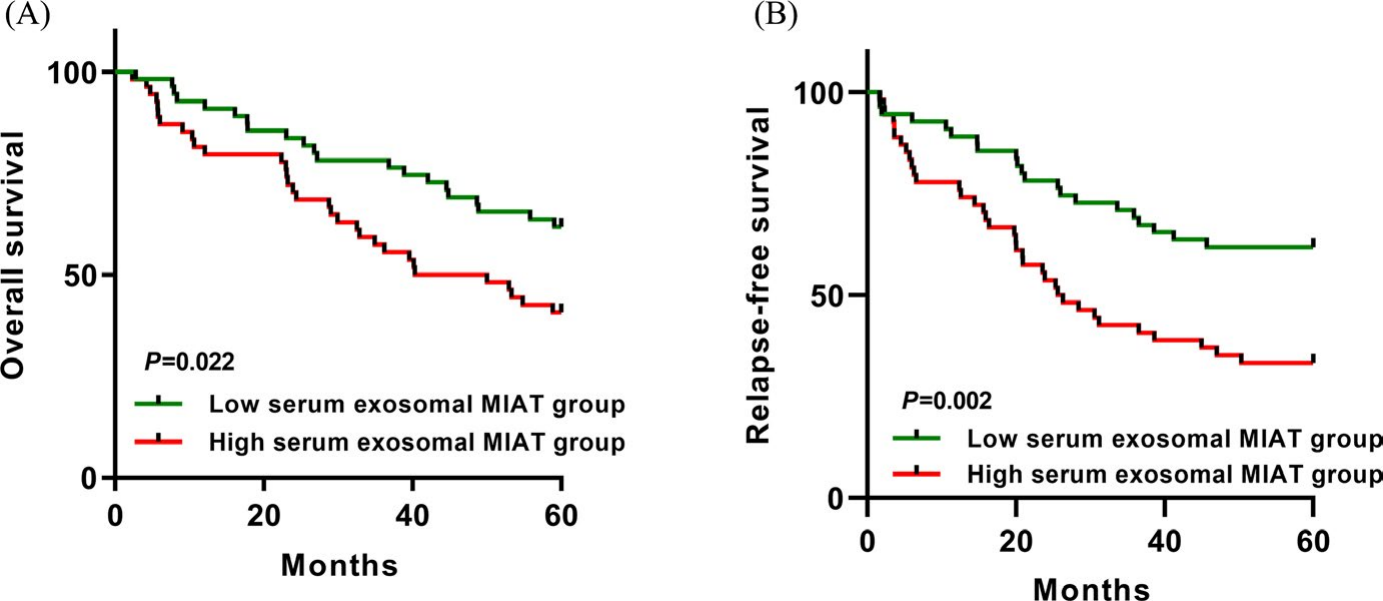
The association between serum exosomal MIAT level and survival of GC. A, GC patients in the high serum exosomal MIAT group suffered significantly shorter OS than those in the low serum exosomal MIAT group. B, GC patients in the high serum exosomal MIAT group had worse RFS than those in the low serum exosomal MIAT group

The survival analysis showed that the GC patients in high serum exosomal MIAT expression group had shorter OS than those in low‐high serum exosomal MIAT expression group (*P* = .022, Figure 4A). Similarly, GC patients with higher serum exosomal MIAT levels suffered worse RFS compared to those with lower serum exosomal MIAT levels (*P* = .002, Figure 4B).

### 3.5 Serum exosomal MIAT was an independent prognostic factor for GC

The multivariate analysis showed that serum exosomal MIAT expression (HR = 3.46, 95% CI = 1.46‐5.69, *P* = .007), differentiation (HR = 2.73, 95%CI = 1.25‐4.47, *P* = .014), lymphatic metastasis (HR = 3.12, 95%CI = 1.37‐5.18, *P* = .011), and TNM stage (HR = 4.07, 95%CI = 1.62‐6.72, *P* = .002) were independent prognostic factors for GC (Table 2).

**Table 2:** Multivariate Cox regression analysis for the relationship between serum exosomal lncRNA MIAT and OS of GC patients

Variables	Hazard Ratio	95% CI	*P*‐value
Differentiation			
Poor vs Well/moderate	2.73	1.25‐4.47	.014
Lymphatic metastasis			
Positive vs Negative	3.12	1.37‐5.18	.011
TNM stage			
III/IV vs I/II	4.07	1.62‐6.72	.002
Serum exosomal lncRNA MIAT			
High vs Low	3.46	1.46‐5.69	.007

## 4 DISCUSSION

GC is one of leading cause of cancer‐related deaths worldwide and monitoring the progression of GC effectively is useful for reducing morbidity and mortality. To the best of our knowledge, this is the first report to analyze prognostic significance of serum exosomal MIAT in GC. The results showed that the exosomes we extracted from the serum samples were positive for exosome markers. Serum exosomal MIAT expression was significantly higher in GC patients. In addition, serum exosomal MIAT well‐differentiated GC cases from gastric adenoma patients and healthy controls. Interestingly, gastric adenoma patients with higher serum exosomal MIAT expression were more prone to progress into GC. Moreover, serum exosomal MIAT levels were significantly decreased in post‐treatment blood samples compared to pre‐treatment samples. Furthermore, serum exosomal MIAT upregulation was correlated with worse clinical variables and shorter OS/RFS. Finally, serum exosomal MIAT was identified as an independent prognostic factor for OS in GC patients. These data suggest that serum exosomal lncRNA MIAT might serve as a promising diagnostic and prognostic biomarker for GC.

MIAT has also been widely reported to play a tumor‐promoting role in many other cancer types. For instance, MIAT was significantly higher in breast cancer (BC) cell lines and high‐grade tumors. Reduced MIAT expression dramatically attenuated cancer cell proliferation, inhibited epithelial‐mesenchymal transition (EMT) and stimulated apoptosis.^18^, ^19^ Upregulation of MIAT was found both in colorectal cancer tissues and cell lines. Knockdown of MIAT markedly suppressed the tumorigenesis through the miR‐132/Derlin‐1 axis.^20^ MIAT was significantly elevated in non‐small cell lung cancer (NSCLC) compared to adjacent normal tissues. MIAT upregulation was strongly correlated with aggressive clinical variables and worse survival. MIAT inhibition dramatically suppressed the malignant behaviors of NSCLC cells and vice versa.^21^, ^22^, ^23^ MIAT was overexpressed in melanoma tissue samples, and enforced MIAT expression significantly promoted the oncogenic activities of cancer cells through the PI3K/AKT signaling pathway.^24^ In hepatocellular carcinoma (HCC), MIAT was highly expressed in HCC tissues than normal tissues. MIAT overexpression or miR‐214 inhibition markedly stimulated HCC cell proliferation and invasion in vivo.^25^ In osteosarcoma, MIAT occurred more frequently in cancerous tissues, and MIAT suppression significantly inhibited cancer cell proliferation.^26^ Similarly, MIAT upregulation was observed in both papillary thyroid cancer (PTC) tissues and cell lines, and its overexpression was closely associated with advanced tumor stage and lymph node metastasis. Reduced MIAT expression remarkably restrained proliferation and invasion in vitro and inhibited tumor growth in vivo.^27^, ^28^ Wang et al showed that MIAT was significantly increased in acute myeloid leukemia (AML) patients and cell lines. MIAT upregulation was correlated with unfavorable clinical outcome of AML.^29^

One limitation of our study was that the sample size was relatively small, and large cohort studies are needed to confirm the diagnostic and prognostic efficacy of serum exosomal lncRNA MIAT for GC. In addition, whether combination of the serum exosomal lncRNA MIAT and currently known tumor biomarkers for GC contribute to predicting survival and recurrence as well as monitoring therapeutic responses warrants further exploration. Moreover, the role of exosomal lncRNA MIAT in tumorigenesis of GC needs further investigation.

## 5 CONCUSSION

Taken together, our study has demonstrated that serum exosomal lncRNA MIAT levels are abnormally upregulated in GC. High serum exosomal lncRNA MIAT level is associated with aggressive clinical parameters and worse prognosis. Therefore, serum exosomal lncRNA MIAT might serve as a promising novel biomarker for monitoring the progression of GC.

## References

[1] Rawla P , Barsouk A . Epidemiology of gastric cancer: global trends, risk factors and prevention. Prz Gastroenterol. 2019;14(1):26‐38. 30944675 10.5114/pg.2018.80001 PMC6444111

[2] Bray F , Ferlay J , Soerjomataram I , Siegel RL , Torre LA , Jemal A . Global cancer statistics 2018: GLOBOCAN estimates of incidence and mortality worldwide for 36 cancers in 185 countries. CA Cancer J Clin. 2018;68(6):394‐424. 30207593 10.3322/caac.21492

[3] Song Z , Wu Y , Yang J , Yang D , Fang X . Progress in the treatment of advanced gastric cancer. Tumour Biol. 2017;39(7):1010428317714626. 28671042 10.1177/1010428317714626

[4] Yao RW , Wang Y , Chen LL . Cellular functions of long noncoding RNAs. Nat Cell Biol. 2019;21(5):542‐551. 31048766 10.1038/s41556-019-0311-8

[5] Zhang X , Wang W , Zhu W , et al. Mechanisms and functions of long non‐coding RNAs at multiple regulatory levels. Int J Mol Sci. 2019;20(22):5573. 10.3390/ijms20225573 PMC6888083 31717266

[6] Perry RB , Ulitsky I . The functions of long noncoding RNAs in development and stem cells. Development. 2016;143(21):3882‐3894. 27803057 10.1242/dev.140962

[7] Yan K , Tian J , Shi W , Xia H , Zhu Y . LncRNA SNHG6 is associated with poor prognosis of gastric cancer and promotes cell proliferation and EMT through epigenetically silencing p27 and sponging miR‐101‐3p. Cell Physiol Biochem. 2017;42(3):999‐1012. 28683446 10.1159/000478682

[8] Zhou R , Wu Z , Deng X , Chen H . The long non‐coding RNA OLC8 enhances gastric cancer by interaction with IL‐11. J Clin Lab Anal. 2019;33(8):e22962. 31273847 10.1002/jcla.22962 PMC6805327

[9] Mo X , Wu Y , Chen L , et al. Global expression profiling of metabolic pathway‐related lncRNAs in human gastric cancer and the identification of RP11‐555H23.1 as a new diagnostic biomarker. J Clin Lab Anal. 2019;33(2):e22692. 30320481 10.1002/jcla.22692 PMC6818562

[10] Gao Y , Chen J . Low expression of lncRNA NBAT‐1 promotes gastric cancer development and is associated with poor prognosis. J BUON. 2019;24(2):656‐662. 31128020

[11] Boukouris S , Mathivanan S . Exosomes in bodily fluids are a highly stable resource of disease biomarkers. Proteomics Clin Appl. 2015;9(3–4):358‐367. 25684126 10.1002/prca.201400114 PMC5502131

[12] Jiang N , Pan J , Fang S , et al. Liquid biopsy: Circulating exosomal long noncoding RNAs in cancer. Clin Chim Acta. 2019;495:331‐337. 31054913 10.1016/j.cca.2019.04.082

[13] Cai C , Zhang H , Zhu Y , et al. Serum exosomal long noncoding RNA pcsk2‐2:1 as a potential novel diagnostic biomarker for gastric cancer. Onco Targets Ther. 2019;12:10035‐10041. 31819499 10.2147/OTT.S229033 PMC6883939

[14] Li S , Zhang M , Zhang H , et al. Exosomal long noncoding RNA lnc‐GNAQ‐6:1 may serve as a diagnostic marker for gastric cancer. Clin Chim Acta. 2020;501:252‐257. 31730812 10.1016/j.cca.2019.10.047

[15] Ishii N , Ozaki K , Sato H , et al. Identification of a novel non‐coding RNA, MIAT, that confers risk of myocardial infarction. J Hum Genet. 2006;51(12):1087‐1099. 17066261 10.1007/s10038-006-0070-9

[16] Sha M , Lin M , Wang J , et al. Long non‐coding RNA MIAT promotes gastric cancer growth and metastasis through regulation of miR‐141/DDX5 pathway. J Exp Clin Cancer Res. 2018;37(1):58. 29540201 10.1186/s13046-018-0725-3 PMC5852965

[17] Li Y , Wang K , Wei Y , et al. lncRNA‐MIAT regulates cell biological behaviors in gastric cancer through a mechanism involving the miR‐29a‐3p/HDAC4 axis. Oncol Rep. 2017;38(6):3465‐3472. 29039602 10.3892/or.2017.6020

[18] Luan T , Zhang X , Wang S , et al. Long non‐coding RNA MIAT promotes breast cancer progression and functions as ceRNA to regulate DUSP7 expression by sponging miR‐155‐5p. Oncotarget. 2017;8(44):76153‐76164. 29100300 10.18632/oncotarget.19190 PMC5652694

[19] Alipoor FJ , Asadi MH , Torkzadeh‐Mahani M . MIAT lncRNA is overexpressed in breast cancer and its inhibition triggers senescence and G1 arrest in MCF7 cell line. J Cell Biochem. 2018;119(8):6470‐6481. 29345338 10.1002/jcb.26678

[20] Liu Z , Wang H , Cai H , et al. Long non‐coding RNA MIAT promotes growth and metastasis of colorectal cancer cells through regulation of miR‐132/Derlin‐1 pathway. Cancer Cell Int. 2018;18:59. 29686537 10.1186/s12935-017-0477-8 PMC5902964

[21] Zhao HL , Xu SQ , Li Q , Zhao YB , Li X , Yang MP . Long noncoding RNA MIAT promotes the growth and metastasis of non‐small cell lung cancer by upregulating TDP43. Eur Rev Med Pharmacol Sci. 2019;23(8):3383‐3389. 31081093 10.26355/eurrev_201904_17702

[22] Lai IL , Yang CA , Lin PC , et al. Long noncoding RNA MIAT promotes non‐small cell lung cancer proliferation and metastasis through MMP9 activation. Oncotarget. 2017;8(58):98148‐98162. 29228680 10.18632/oncotarget.21465 PMC5716720

[23] Lin D , Xu HP , Lin JH , Hu HH , Wang Q , Zhang J . Long non‐coding RNA MIAT promotes non‐small cell lung cancer progression by sponging miR‐1246. Eur Rev Med Pharmacol Sci. 2019;23(13):5795‐5801. 31298331 10.26355/eurrev_201907_18318

[24] Yang Y , Zhang Z , Wu Z , Lin W , Yu M . Downregulation of the expression of the lncRNA MIAT inhibits melanoma migration and invasion through the PI3K/AKT signaling pathway. Cancer Biomark. 2019;24(2):203‐211. 30614798 10.3233/CBM-181869 PMC13082490

[25] Huang X , Gao Y , Qin J , Lu S . lncRNA MIAT promotes proliferation and invasion of HCC cells via sponging miR‐214. Am J Physiol Gastrointest Liver Physiol. 2018;314(5):G559‐G565. 29097358 10.1152/ajpgi.00242.2017

[26] Jin H , Jin X , Chai W , et al. Long non‐coding RNA MIAT competitively binds miR‐150‐5p to regulate ZEB1 expression in osteosarcoma. Oncol Lett. 2019;17(1):1229‐1236. 30655889 10.3892/ol.2018.9671 PMC6312950

[27] Wang R , Zhao L , Ji L , Bai L , Wen Q . Myocardial infarction associated transcript (MIAT) promotes papillary thyroid cancer progression via sponging miR‐212. Biomed Pharmacother. 2019;118:109298. 31404776 10.1016/j.biopha.2019.109298

[28] Liu W , Wang Z , Wang C , Ai Z . Long non‐coding RNA MIAT promotes papillary thyroid cancer progression through upregulating LASP1. Cancer Cell Int. 2019;19:194. 31372094 10.1186/s12935-019-0913-z PMC6659215

[29] Wang G , Li X , Song L , Pan H , Jiang J , Sun L . Long noncoding RNA MIAT promotes the progression of acute myeloid leukemia by negatively regulating miR‐495. Leuk Res. 2019;87:106265. 31698307 10.1016/j.leukres.2019.106265

